# The efficacy of biosynthesized silver nanoparticles against *Pseudomonas aeruginosa* isolates from cystic fibrosis patients

**DOI:** 10.1038/s41598-023-35919-6

**Published:** 2023-06-01

**Authors:** Hafez Al-Momani, Muna Almasri, Dua’A. Al Balawi, Saja Hamed, Borhan Aldeen Albiss, Nour Aldabaibeh, Lugain Ibrahim, Hadeel Albalawi, Sameer Al Haj Mahmoud, Ashraf I. Khasawneh, Muna Kilani, Muneef Aldhafeeri, Muayyad Bani-Hani, Matthew Wilcox, Jeffrey Pearson, Christopher Ward

**Affiliations:** 1 Department of Microbiology, Pathology and Forensic Medicine, Faculty of Medicine, Hashemite University Medical School, The Hashemite University, Zarqa, 13133 Jordan; 2 Faculty of Applied Medical Sciences, The Hashemite University, Zarqa, 13133 Jordan; 3 Department of Pharmaceutical and Pharmaceutical Technology, Faculty of Pharmaceutical Sciences, The Hashemite University, Zarqa, Jordan; 4 Nanotechnology Institute, Jordan University of Science & Technology, P.O. Box 3030, Irbid, 22110 Jordan; 5 Supervisor of Microbiology Laboratory, Laboratory Medicine Department, Jordan University Hospital, Amman, Jordan; 6 Department of Basic Medical Science, Faculty of Medicine, Al-Balqa’ Applied University, AL-Salt, Jordan; 7 Department of Pediatrics, Faculty of Medicine, The Hashemite University, Zarqa, Jordan; 8 Institutes of Cellular Medicine and Cell & Molecular Biosciences, Newcastle University Medical School, Newcastle University, Newcastle Upon Tyne, NE2 4HH UK; 9 Department of Plant Production and Protection, Faculty of Agriculture, Jerash University, Jerash, Jordan; 10 Biosciences Institute, Medical School, Newcastle University, Newcastle Upon Tyne, NE2 4HH UK

**Keywords:** Microbiology, Molecular biology, Diseases

## Abstract

The high antibiotic resistance of *Pseudomonas aeruginosa* (PA) makes it critical to develop alternative antimicrobial agents that are effective and affordable. One of the many applications of silver nanoparticles (Ag NPs) is their use as an antimicrobial agent against bacteria resistant to common antibiotics. The key purpose of this research was to assess the antibacterial and antibiofilm effectiveness of biosynthesized Ag NPs against six biofilm-forming clinically isolated strains of PA and one reference strain (ATCC 27853). Ag NPs were biosynthesized using a seed extract of *Peganum harmala* as a reducing agent. Ag NPs were characterized by Ultraviolet–visible (UV–Vis) spectroscopy and scanning transmission electron microscopy (STEM). The effect of Ag NPs on biofilm formation and eradication was examined through micro-titer plate assays, and the minimal inhibitory (MIC) and minimum bactericidal (MBC) concentrations determined. In addition, real-time polymerase chain reactions (RT-PCR) were performed to examine the effects of Ag NPs on the expression of seven PA biofilm-encoding genes (*LasR, LasI, LssB, rhIR, rhII, pqsA* and *pqsR*). The biosynthesized Ag NPs were spherically-shaped with a mean diameter of 11 nm. The MIC for each PA strain was 15.6 µg/ml, while the MBC was 31.25 µg/ml. All PA strains exposed to Ag NPs at sub-inhibitory concentrations (0.22–7.5 µg/ml) showed significant inhibitory effects on growth and biofilm formation. Biomass and biofilm metabolism were reduced dependent on Ag NP concentration. The expression of the quorum-sensing genes of all strains were significantly reduced at an Ag NP concentration of 7.5 µg/ml. The results demonstrate the extensive in-vitro antibacterial and antibiofilm performance of Ag NPs and their potential in the treatment of PA infection. It is recommended that future studies examine the possible synergy between Ag NPs and antibiotics.

## Introduction

*Pseudomonas aeruginosa* (PA) is a common nosocomial pathogen that can cause death in those with immunosuppression, malignancy, burns, traumatic wounds and cystic fibrosis^1^. PA can form a biofilm on various abiotic surfaces, including artificial implants, urinary catheters, endotracheal tubes, and contact lenses^2^. Extracellular polymeric substances (EPS) form the matrix-like structures of biofilms that surround the bacterial communities^3^. Biofilms are a significant concern as they can resist host immune systems and many antimicrobials^3^. Antimicrobial peptides are electrostatically repulsed from or broken down by the biofilm matrix, shielding the cells inside from phagocytosis and the host’s immune response^4,5^. A cell-to-cell communication system called quorum sensing (QS) regulates several processes in PA, including biofilm production^6^.

The target cells of many antimicrobials lie deeply entrenched within the biofilm matrix, making their treatment extremely difficult^7^. Antibiotic resistance is now a major global health concern^8^, and the need for non-antibiotic treatments for drug-resistant microbial illnesses has increased significantly. Nanoparticles offer an alternative, easy to synthesize, approach to the treatment of PA infections as their small size and high surface-to-volume ratio makes them effective against biofilms^9^.

Of many commonly produced nanoparticles, Ag NPs stand out as having outstanding ability to fight pathogenic multidrug-resistant bacterial isolates^10^. It is widely acknowledged that Ag NPs have antibacterial and antibiofilm activity on both non-resistant and resistant pathogenic bacteria^10–12^. Ag NPs exert their antibiofilm activity by recognizing the peptidoglycan structure of bacterial membranes and binding to the exopolysaccharide matrix. The biofilm structure is then severely damaged or destroyed through the oxidative stress and DNA damage produced by ROS production and ion release^13–15^. Ag NPs also have significant potential for use in medical and and other non-medical uses due to their wide size range, ability to self-assemble capacity, and high antibacterial activity^16^.

Biogenic-mediated nanotechnology incorporates green chemistry principles in the production of nanoparticles. Plant extracts have been widely studied in the manufacture of metallic nanoparticles and can increase their monodispersity. The biomolecules found in plants (e.g. flavones, phenolics, proteins, polysaccharides, terpenoids, alkaloids, enzymes, amino acids, and alcohols) are effective reducing agents and serve as capping agents that stabilize and control the NPs' structure^17–20^. Biogenic techniques provide a clean and eco-friendly process for synthesizing the nanoparticles used in many cosmetic and pharmaceutical applications. Thus, there is widespread interest in the application of biogenic techniques for the green production of nanoparticles^21,22^.

The glabrous and perennial plant *Peganum harmala*, commonly known as Harmal, grows in sandy soils in semi-arid areas. The shrub measures between 0.3 and 0.8 m in length and contains spherical seed capsules with more than 50 seeds and white blooms. In the Middle East, this plant is widely cultivated for medicinal uses and found in the marginal and desert regions in Jordan^23^ where it has traditionally been used as an antiseptic and disinfectant by burning or boiling its seeds^24^. The plant has been used for the treatment of a variety of human ailments including lumbago, asthma, colic, jaundice, and as a stimulating emmenagogue^25,26^. It has also been claimed to have antibacterial, antiviral, and antifungal properties^26,27^.

In the current study, *P harmala* was used as a reducing agent in the biosynthesis of Ag NPs. The antibacterial and antibiofilm properties of the Ag NPs were examined on clinical isolates of six PA strains. The effect of exposure to different concentrations of Ag NPs during biofilm formation were also assessed. In addition, the influence of Ag NPs on the expression of genes involved in QS regulation and the polysaccharide synthesis of PA biofilm was investigated.

## Method

### Bacterial isolates

The American Type Culture Collection (ATCC) PA 27853 standard strain, as well as six clinical isolates (PA1, PA2, PA3, PA4, PA5, and PA6) were used in this study. The PA ATCC was obtained from the international PA panel and its entire genome sequence is available^28^. The clinical strains were isolated from sputum samples of patients with cystic fibrosis at the Jordanian Prince Hamza Hospital's microbiology laboratory. Gram-stain, the synthesis of green pigments on nutrient agar, growth on MacConkey agar, the oxidase test, motility, growth on selective medium-cetrimide agar, and the capacity to grow at 42 °C were used to identify the PA bacterium. The VITEK2 computer automatic bacterium identification system (Bio Merière, Lyon, France) was used for verification.

### Culture conditions

The Brain–Heart Infusion (BHI) broth technique was used to enrich the samples (OxoidTM). After enrichment, the samples were grown using the streak plate and pour plate approaches on Pseudomonas Cetrimide Agar (PSA) (OxoidTM). For each strain, the researchers initiated subcultures from single colonies, all of which were cultivated aerobically on PSA plates at 37 °C. After 18–24 h incubation, fresh cultures were prepared at concentrations of 0.5 on the McFarland Scale (1.5 × 10^8^ CFU/mL). Several different dilutions were tested during the assessment process.

### Preparation of aqueous *P. harmala* seed extract

Whole *P. harmala* plants were gathered from the Al-Hallabat area of the Zarqa Governorate in July/2022 from a Ministry of Agriculture farm. The identification of *P. harmala* seeds was checked by comparison with the verified sample held in the herbarium of the Faculty of Agriculture at Jordan University of Science and Technology (JUST) and confirmed by the Assistant Professor of Agriculture (Dr Muayyad Bani-Hani). Voucher specimen (AM/2023/01/001) was deposited at the herbarium located at the Plant Production and Protection Department of Jerash University, Jordan. *P. harmala* were harvested in accordance with all institutional, national, and international guidelines and legislation.

The seeds were washed with double-distilled water (Sigma-Aldrich HPLC Plus Water), thoroughly dried at room temperature, and mechanically ground into a coarse powder. Five grams of this powder were combined with 50 ml of double-distilled water and heated to 70 °C for 15 min before being twice filtered through Whatman filter paper. Micro filtration through a 0.22 mm syringe membrane filter was used to produce a clear aqueous extract with a pH of 5.4 at 25 °C.

### Green synthesis of silver nanoparticles

A 50 ml solution of 3 mM AgNo3 was warmed to 80 ℃ in a foil-covered 100 ml Erlenmeyer flask and mixed using a magnetic stirrer at a continual speed of 1100 rpm. Aqueous *P. harmala* seed extract was added dropwise at a rate of 34 µl/min using a micropipette until 4 ml had been added. A change in the solution color from orange to dark brown was the first indication of Ag NP synthesis. The solution was then centrifuged at 6000 rpm for 30 min three times to eliminate unreacted *P harmala*.

### Characterization of silver nanoparticles

#### UV–visible spectroscopy

UV–visible spectroscopy (UV-1900, SHIMADZU, Japan) was used to visually track the bio reduction of the AgNO3 and production of biogenic Ag NPs from the aqueous *P. harmala* extract. Double-distilled water was used as the control reference. Each spectrophotometric analyses was performed in a quartz cuvette with a path length of 1.00 cm. The surface plasmon resonance (SPR) of the Ag NPs was measured using a UV–visible spectrophotometer, using wavelengths of 800–300 nm as a marker of Ag NP formation.

#### Scanning Transmission Electron Microscope (STEM)

The scanning transmission electron microscope (STEM) (Versa 3D, FEI, Netherlands) produces images derived from electrons passing through a thin specimen. This was used to test the morphology and size characteristics for the synthesized silver nanoparticles.

#### X-ray diffraction

An X-ray diffractometer was used in order to measure the X-ray diffraction (XRD) spectra of the zinc oxide NPs. This was equipped with a radiation source comprising CuKα, which had a 0.154 nm wavelength. The specimen container measured 2 cm × 0.5 mm.

#### Fourier-Transform Infrared Spectroscopy (FT-IR)

FT-IR analysis of the Ag NPs and *P. harmala* seed extract was performed in Hashemite University’s chemistry department and enabled the relationships between functional groups of the *P. harmala* extract and the synthesized Ag NPs to be evaluated within a wavenumber range of 3600–400 cm^−1^.

### Testing the antibiotic susceptibility of clinical isolates

Disc diffusion was employed to evaluate the susceptibility of the PA strains to 10 anti-pseudomonal antibiotics according to the Clinical Laboratory and Standards Institute's (CLSI) procedure. Cell suspension in saline was adjusted to 0.5 McFarland and inoculated in Muller Hinton Agar-MHA to initiate bacterial exponential growth (18–24 h) (Sigma-Aldrich). The plate was covered with antibiotic discs, which were then incubated for 18–24 h at 35 ± 2 °C. Bacterial susceptibility to these antibiotics was confirmed by measuring the diameter of the inhibition zones created and interpreting them in accordance with CLSI set values^29^.

Ten different anti-pseudomonal antibiotics were used: aztreonam 30 µg (ATM), ciprofloxacin 5 µg (CIP), piperacillin 100 µg (PRL), amikacin 30 µg (AK), ceftazidime 30 µg (CAZ), cefepime 30 µg (FEP), gentamicin 10 µg (CN), levofloxacin 5 µg (LEV), imipenem 10 µg (IPM) and meropenem 10 µg (CT). All anti-pseudomonal discs were purchased from Oxoid, UK.

### Minimal Inhibitory Concentration (MIC) Test

The MIC of an antimicrobial agent refers to the minimum concentration required to suppress visible growth of the target microbes after 12 h’ incubation. The minimum bactericidal concentration (MBC) refers to the minimum concentration needed to ensure no microbes remain viable after exposure to the antimicrobial (i.e. that no growth is observed in subsequent culture in untreated media).

The MIC for the Ag NPs was determined using the microtiter broth dilution process^30^. The Ag NPs were employed against the ATCC reference strain PA 27853 and the six clinical isolates in triplicate and in three separate experiments. In each test, 100 μl of bacteria at a density of 5 × 10^5^ CFU/ml in Muller Hinton Broth (MHB) (Oxoid) were added to the 96-well assay plates (Corning, NY) containing different Ag NP concentrations. PA ATCC 27853 was used as the positive control while the negative control was inoculated broth incubated under identical conditions but without addition of Ag NP.

Ag NP concentrations used for testing ranged from 1 mg/ml to 3.9 μg/ml and were obtained through two-fold serial dilution. The inoculated microplates underwent a 24-h incubation period at 37 °C and 150 rpm. The control was incubated for 24 h at 37 °C and contained inoculated broth with no Ag NPs. The MIC endpoint refers to the minimum Ag NP concentration at which no discernible growth could be observed in the wells. Tetrazolium-based microtiter dilution was used for confirmation.

As cellular NADH drives the creation of colored formazan salts, tetrazolium salts are frequently used as reagents in biological tests to assess the metabolic behaviour of living cells^31^. Tetrazolium-based dyes are therefore commonly used to identify MICs and evaluate biofilms^32^. In this study triphenyl tetrazolium chloride (TTC) was used as a metabolic marker of bacterial survival and biofilm development. After the initial 24 h incubation, 40 μl of 0.2 mg/ml TCC dissolved in deionized water was added to the wells and incubation continued for a further 4–6 h at 37 °C. The color changes were then assessed and compared to those of the controls.

The lowest concentration not changing the color of the dye was considered as the MIC against the target bacteria. To attain reliability the experiment was repeated three times each with both positive and negative controls.

After establishing the Ag NP MICs, 50 µl aliquots were seeded from all wells showing no obvious bacterial growth. They were placed on BHI agar plates and incubated at 37 °C for 24 h. Subsequently, the elimination of 99.9% of the bacteria by the lowest concentration of Ag NPs was taken as the MBC endpoint (Supplementary Fig. 1).

### Bacterial growth assay

Sterile and untreated clear microtiter plates with 96-well flat bottoms (BD Falcon)^33^ were employed to examine bacterial growth and biofilm development under the different Ag NP concentrations. To perform the growth test, standard suspensions were created of each PA culture. Aliquots of 105 µl Tryptone Soy Broth (TSB) were applied to each well, to which 20 µl aliquots of bacteria were added and combined with 125 µl of the different concentrations of Ag NPs (0.225–7.5 µg/ml). This gave a total volume of 250 µl in each well of the microtiter tray. All assays were carried out three times. Positive and negative controls were also used, with the former containing PA and TSB (and no Ag NPs) and the latter only containing TSB.

After the preparation was completed, the cultures underwent aerobic incubation for 24 h at 37 °C, during which they were stored in darkness and were subjected to shaking. Following the incubation, the cell numbers at different concentrations of Ag NPs were determined by evaluating the optical density (OD) at 600 nm (Infinite^®^ 200 PRO NanoQuant, TECAN). The final cell growth value for each Ag NP concentration was calculated as the difference between the averages of the well readings with and without PA inoculation. All cells exhibited biofilm-associated and planktonic cell expansion.

### Quantitative determination of biofilm formation via microtiter-plate test

A spectrophotometric approach was used to quantify biofilm production by measuring its total biomass—which includes bacterial cells and EPS^34,35^. Two distinct wells were created on separate microtiter plates in parallel for each test condition. One of the wells was then stained with Crystal Violet (CV) and the other with the metabolic TTC dye.

After incubating the plate for 24 h without shaking (much like a growth experiment), each well was aspirated and washed three times in sterile physiological saline (250 µl). After the well was aspirated the plates were vigorously shaken to remove any unbound bacteria.

The remaining microorganisms on the first plate were fixed in 200 µl of 99% methanol. The plates were left for 15 min, after which they were emptied and allowed to dry. Each plate was then stained for 5 min with 2% Hucker CV, a Gram-stain-safe solution, applied at 200 µl to each well. To remove any extra staining material the wells were cleaned three times in 200 µl of sterile water. Care was taken not to disrupt the biofilm during the wash stage. The plates were then left again to dry before the cell-bound dye was resolubilized using 160 µl of 33% (v/v) glacial acetic acid per well.

An automated reader (the ICN Flow Titertek Multiscan Plus) was used to measure the OD measurements in the wells. Readings were taken at three different times: first, before the samples were incubated (OD 600 nm); second, post-incubation to evaluate growth (OD 600 nm); and third, after completing the biofilm test (OD 570 nm). A ratio of 570/600 was used to normalize the measurement of biofilm formation against bacterial growth. Negative OD values were displayed as zero. Each experiment was performed three times and a cut-off value (ODc) determined from the standard deviations (SDs). The ODc used was the mean of the negative control + 3 SDs. The isolates were then assigned to four biofilm producer categories as follows: non producer (OD < ODc); weak producer (ODc < OD < 2 × ODc); moderate producer (2 × ODc < OD < 4 × ODc); and strong producer (4 × ODc < OD).

Metabolic activity was assessed on the second plate with TTC used as a marker for viable bacterial growth. TTC is converted by metabolically active cells into a formazan derivative that is easy to measure colorimetrically. Similar to growth experiments, the plates were vigorously shaken to remove any bacteria that had not adhered after incubation for 24 h at 37 °C.

All wells had their media removed after incubation and 200 μl of phosphate-buffered saline (PBS) was used to wash the biofilm. The biofilm cells from the wells with biofilm were vigorously pipetted into the suspension after adding 100 μl of PBS solution. The suspended biofilm was then moved onto a fresh 96-well flat bottom microplate.

A final concentration of 0.02% was obtained by adding 50 μl of 0.1% TTC (Sigma, USA). The samples were left to incubate at 37 °C for 4–5 h after which the OD405 was measured with a VERSA max microplate reader. Colorimetric absorbance at 405 nm is an effective means to quantify the inhibition of bacterial growth as red formazan is produced when viable bacteria reduce TTC^36^.

### Production of extracellular polymeric substance (EPS) in the presence of Ag NPs

To evaluate the impact of Ag NPs on the EPS production, a modified ethanol precipitation method^37^ was used. EPS extraction of each strain was performed in triplicate. A 0.5 McFarland bacterial suspension of each strain in LB broth was prepared and added in 100 ml of LB broth having Ag NPs at concentrations range from 7.5 to 0.225 μg/ml and were incubated at 37 °C for 24 h. Control was prepared without the addition of Ag NPs. Following incubation, EPS was extracted from bacterial cultures by centrifuging at 10,000 rpm for 30 min at 4 °C. To precipitate the EPS, 1 volume of 95% ethanol was added to each 3 volumes of the supernatant collected from the centrifuge and placed under refrigeration at 4 °C for 18 h. The mixture was then again centrifuged at 10,000 rpm for 20 min while maintaining the temperature at 4 °C. The precipitated EPS was collected and put back into suspension using double-distilled water. Any traces of bacterial cells were removed from the suspension by filtration using a 0.45 μm cellulose acetate membrane. To confirm the absence of any viable bacteria, a sample was placed on an agar plate under optimal culture conditions to check for growth. The extracted EPS was then lyophilized and weighed to give a result in mg EPS per 100 ml.

### Microtiter Biofilm Eradication Assay

A technique combining CV staining^34,35^ with a metabolism-sensitive tetrazolium dye was used to assess the impact of Ag NPs on pre-formed biofilms. Standardized bacterial suspensions were created by overnight culture in two 96-well plates with TSB at a concentration of 0.5 McFarland. Biofilms were produced by adding 20 µl aliquots of bacterial suspension to 250 µl aliquots of TSB (with no Ag NPs). The plates were then left to incubate overnight at 37 °C to enable the biofilm to grow and attach to the plates.

Two separate wells were created for each condition on individual microtiter plates, one for staining with CV and the other for staining with metabolic TTC. The media was taken off the plate the following day and left to dry for 15 min on a paper towel at room temperature. After the planktonic and unattached cells were removed, a multi-channel pipette was used to rinse the adhering biofilm. This process was performed three times using 150 µl of sterile media. Each well had its excess washing medium aspirated before adding 200 µl of fresh MHB containing the different concentrations of Ag NPs. The plates were incubated at 37 °C for 24 h. Planktonic growth was examined the next day, after which the planktonic cells and used media were removed, and the residual biomass washed three times in distilled water. One plate was then stained with CV and the other with TCC. Average OD490 values were then obtained from the findings of the three independent biological replicates^36^.

### Genotypic detection of Ag NP inhibitory activity using quantitative real-time PCR

A qRT-PCR test was performed to estimate the impact of high (7.5 µg/ml) and low (0.45 µg/ml) concentrations of Ag NPs on the expression of QS-regulatory genes. PA was incubated in MH medium with the Ag NPs at 37 °C and 50–60 rpm in 6-well plates (Corning, NY). Controls were incubated under identical conditions but without Ag NPs. After exposure for 24–48 h (exponential phase of biofilm growth), the biofilm was carefully harvested and gently rinsed with 10 mM NaCl to remove unattached cells. Biofilm RNA was extracted using RNeasy Mini Kit (Qiagen, Germany).

To synthesize the cDNA, random primers (including RNaseOUT, dNTPs, and Superscript II reverse transcriptase (Tsingke, Beijing, China) underwent reverse transcription PCR reaction at 42 °C. Quantitative polymerase chain reactions (qPCR) were conducted using a BIO-RAD Thermal cycler to achieve amplification. Subsequently, 2 μl of template DNA was added to a 0.5 μl solution of each forward and reverse primer, 10 μl of Luna Universal qPCR Master Mix and 7 μl of nuclease free water to give a final reaction volume of 20 μl.

A gradient PCR reaction was conducted for all of the PCR primers used in this study. The cycling conditions were subjected to pre-denaturation at 95 °C for 3 min, after which 34 cycles of denaturation were performed at 94 °C, each for thirty seconds, and annealing at a temperature range between 50 and 63 °C for thirty seconds. This was followed by elongation at 72 °C for 60 s. Finally, the reaction was extended for 5 min at a temperature of 72 °C. The relative expression values of QS-regulatory genes were normalized to the housekeeping genes rpoD and rpsL, and the specific PCR amplification was verified using agarose gel electrophoresis. For the treated PA, the gene expression level was computed relative to that in the untreated PA. To do this, the 2^−ΔΔCt^ technique was employed^38^. The primer sequences used in this research are presented in Supplementary Table 1.

### Cytotoxicity Assay

The HT29-MTX cell line was used to assess the cytotoxicity of Ag NPs using methyl tetrazolium (MTT) assay^39^. HT29-MTX originates from the goblet cells of the colon but secretes the mucin MUC5AC, characteristic of the goblet cells found in respiratory system cells, rather than the MUC2 mucin characteristic of the goblet cells in the colon. HT29-MTX cells were cultured in T75 flasks with 10% CO_2_ at 37 °C in 12 ml Dulbeco Modified Eagles Medium (DMEM, Sigma, UK) with the addition of 10% FCS (Sigma, UK), 50 U/ml penicillin, 50 mg/ml streptomycin (Sigma, UK), 50 mg/ml gentamycin (Lonza, USA), and 50 µg/ml amphotericin B (Lonza, USA).

The HT29 MTX cells were then rested for 24 h in serum-free DMEM containing 50U/ml penicillin, 50 mg/ml streptomycin, 50 mg/ml gentamycin, and 50 µg/ml amphotericin B. Ag NPs were then added at concentrations of 7.5–0.225 µg/ml. The Ag NP treated cells and the control cells were incubated for 24 h with 5% CO_2_ at a relative humidity of 95%. The media was then removed and the cells washed using PBS. The wells then had 25 ml of MTT solution (5 mg/ml) added and were incubated for 4 h. The medium was removed and replaced with 100 ml of DMSO and agitated gently for 15 min. To assess the effect of the Ag NPs on cell viability, the enzyme linked immunosorbent assay plate reader was used to measure 570 nm absorbance and the viable cell percentage estimated for the Ag NP-treated and control cells^38^.

### Ethical approval

Hashemite University and Prince Hamza Hospital’s Ethics Service Committee granted ethical approval for this case study (reference number 7/10/2019-2020). All experiments were performed in accordance with relevant guidelines and regulations. The PA clinical isolates from the sputum samples were taken after informed consent was obtained from all CF patients.

### Statistical analysis

The mean (M) and standard error of the mean (SEM) of at least three replicates were used to express the experimental results. One-way analysis of variance (ANOVA) was used to identify differences between the samples and the controls. The OD values from the microtiter-plate experiments (with and without nanoparticles) at various dilutions were compared using Tukey's test. P values under 0.05 were regarded as significant. The data were examined using Graphpad Instat 6.0 software.

## Results

### Characterizing the silver nanoparticles

**Figure 1:**
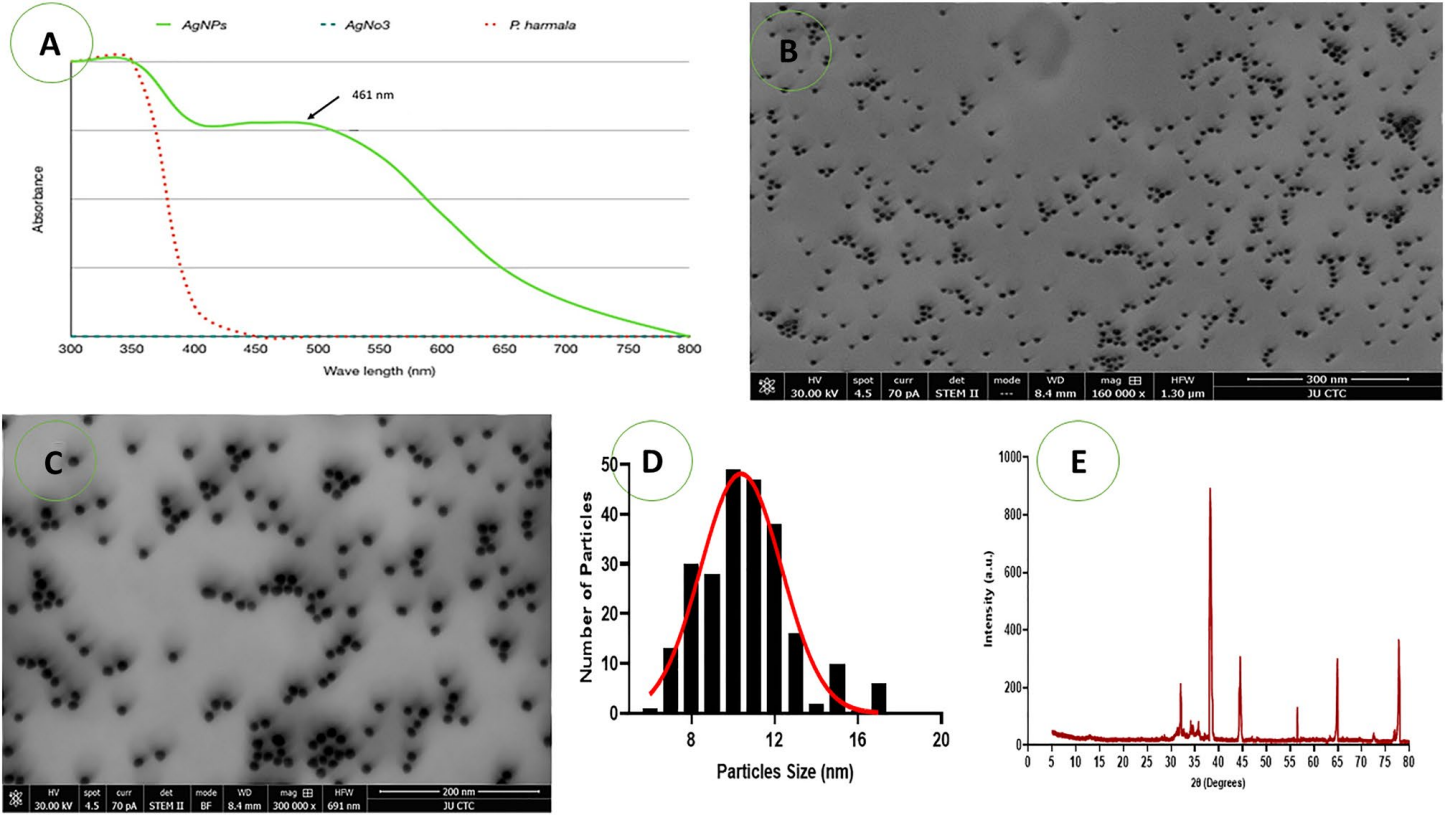
(**A**–**E**) The UV–visible spectrum of synthesized Ag NPs (**A**). The STEM images for synthesized Ag NPs, magnification at 300× (**B**) and at 200 nm (**C**). The size histograms of the Ag NPs (**D**), XRD pattern of synthesized Ag NPs (**E**).

During Ag NPs preparation, the formation of Ag NPs causes a color change in the reaction mixture from orange to dark brown, indicating AgNO_3_ reduction from the excitation of surface plasmon resonance (SPR) in the Ag NPs (Supplementary Fig. 2). The UV–Vis Spectrum (shown in Fig. 1A) reveals a broad absorption peak at around 461 nm, which is consistent with the Ag surface plasmon resonance that occurs between 450 and 500 nm.

**Figure 2:**
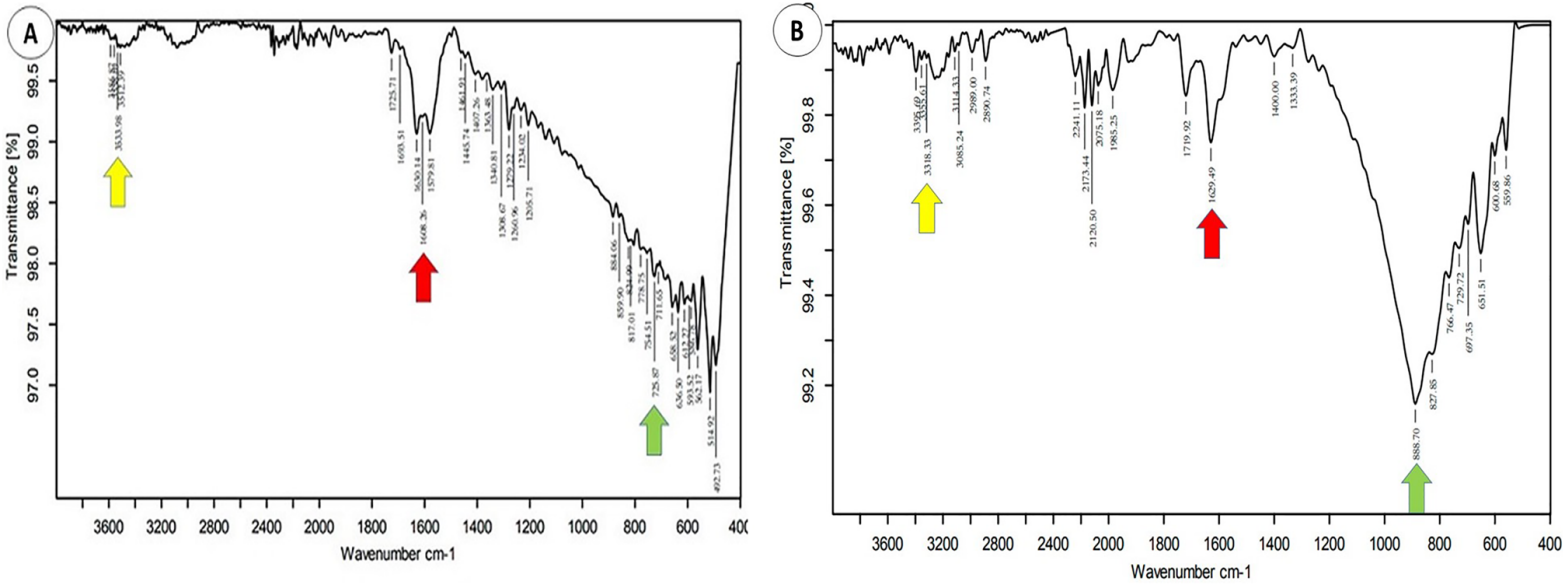
The FT-IR spectra: (**A**) *P. harmala* extract. (**B**) Synthesized Ag NPs.

The STEM image for the Ag NPs that were synthesized using *P. harmala* seed extract can be seen in Fig. 1. The dark field image in Fig. 1B shows that they were biosynthesized Ag NPs with an average particle size of approximately 11 nm. Furthermore, the bright field image for Ag NPs displayed in Fig. 1C highlights the uniformity of the synthesized particles, their spherical shape, and the lack of aggregation. The size histograms of the Ag NPs are shown in Fig. 2D, The histograms indicate that the main particle sizes of the Ag NPs made in this research is about 10.9 ± 2.7 nm.

The XRD pattern of the previously primed silver nanoparticle sample was documented at the UGC-DAE Consortium for Scientific Research, Indore. For this purpose, a Bruker d8 Advance X-ray diffractometer was utilised. The follow parameters were applied: radiation source, CuKα, λ = 1.5406 Å; 40 kV–40 mA; scanning mode, 2θ/θ.

A comparison was made between the diffractogram, shown in Fig. 1, and the JCPDS standard power diffraction card, silver file No. 04-0783. Four peaks were recognised at 2θ values, i.e. 38.2901°, 44.5583°, 64.8185° and 77.4383°, which were deemed to reflect the silver metal and the associated silver plane values of hkl, i.e., (111), (200), (200) and (311), respectively. In Fig. 1D.

FT-IR was used to identify the interactions that take place between Ag atoms and bioactive chemicals which impact the stability of Ag NPs (Fig. 2). Similar FT-IR spectra peaks with a slight shift can be seen in the biosynthesized Ag NPs and the *P. harmala* extracts. In Fig. 2A,B, different arrow colors represent different functional groups. The yellow denotes the peak at 3533.98 cm^−1^ for the *P. harmala* extract spectrum, which changed to 3318.33 cm^−1^ for the Ag NPs-extract spectrum and was assigned to the OH-stretching of hydroxyl groups, including alcohols, phenols, and the NH group of amines or amides. The red represents the peak at 1608.36 cm^−1^ for the *P. harmala* extract spectrum which changed to 1629.49 cm^−1^ for the Ag NPs-extract spectrum and was assigned to the C=O group of carboxylic acids. Finally, the green arrow indicates the peak for the *P. harmala* extract spectrum at 725.87 cm^−1^ which changed to 888.70 cm^−1^ for the Ag NPs-extract spectrum and was assigned to C=CH2.

### Antibiotic susceptibility and resistance profiles of the clinical isolates

Antibiotic susceptibility was evaluated using the disc diffusion approach. Four clinical isolates (PA2, PA4, PA6 and PA5) demonstrated intermediate resistance to one (Gentamicin), two (Imipenem and Levofloxacin), four (Imipenem, Gentamicin, Levofloxacin and Ciprofloxacin) and five of the antibiotics employed in this trial (Meropenem, Gentamicin, Amikacin, Levofloxacin and Ciprofloxacin) respectively. Three clinical isolates (PA3, PA5 and PA6) were found to be resistant to cefepime. However, it is important to note that none of the isolates were multidrug-resistant (MDR). Supplementary Table 2 shows the results of the antibiotic susceptibility tests for ATCC and the clinical strains, with PA5 having the highest resistance to all the studied antibiotics.

### MIC and MBC

After samples had been incubated at 37 °C for 24 h under aerobic conditions and with Ag NPs at concentrations ranging from 3.9 to 1000 μg/ml, turbidity was present in the test tubes containing 3.9–7.8 µg/ml of Ag NPs. This turbidity indicated that bacteria were growing at these Ag NP concentrations. However, no turbidity could be seen in concentrations of 15.6–1000 µg/ml, indicating that bacterial growth had been inhibited. BHI agar plates were inoculated with the suspensions from the tubes of 15.6–1000 µg/ml for a period of 24 h. No bacteria could be seen at concentrations of 31.25–1000 µg/ml, verifying that the Ag NPs were bactericidal. Thus, from these findings it is evident that the MIC and MBC of Ag NPs for all PA strains were 15.6 and 31.25 µg/ml, respectively.

### The inhibitory effect of silver on planktonic growth and biofilm formation of PA

**Figure 3:**
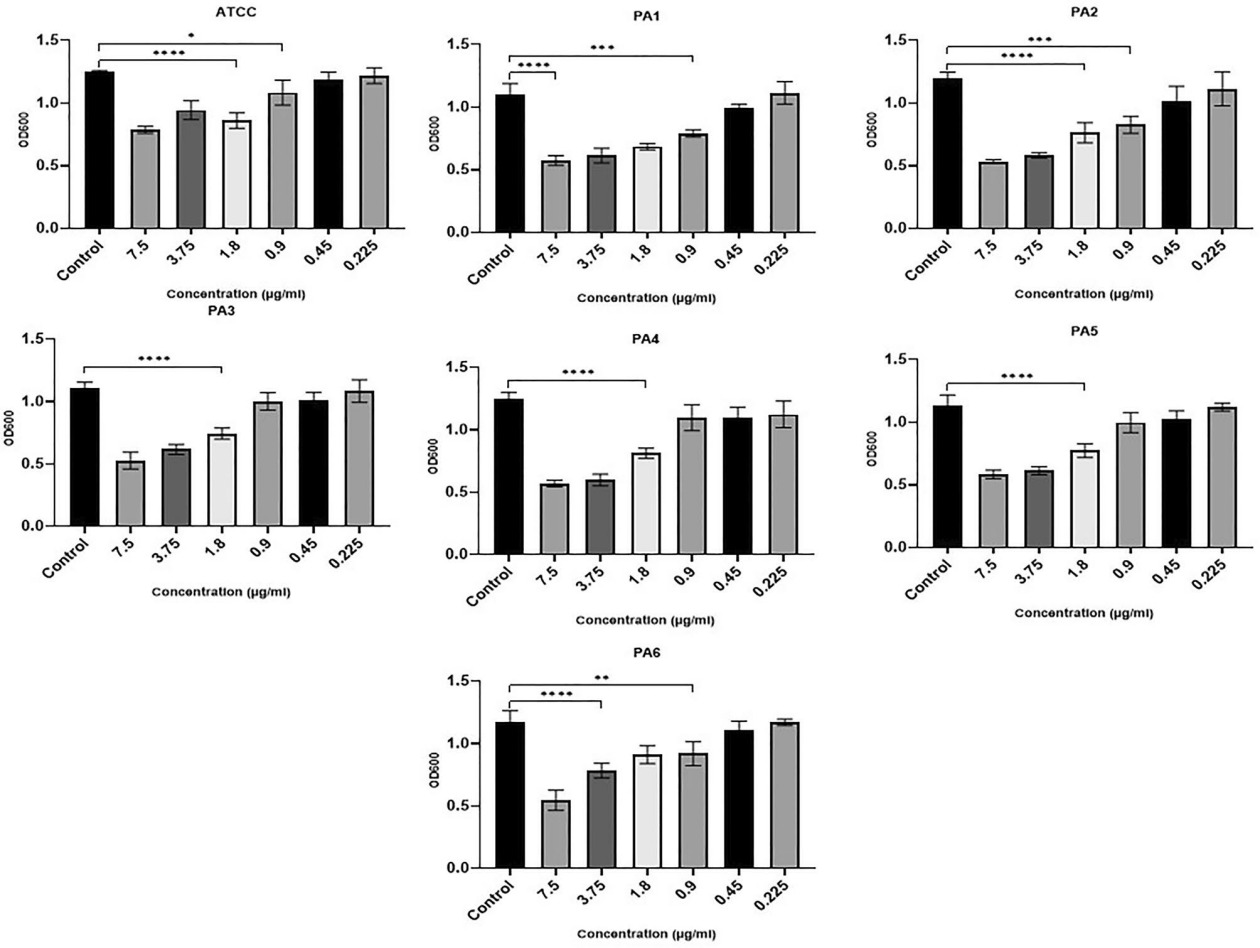
The impact of Ag NPs on PA planktonic growth following 24 h of incubation indicated by absorbance at 600 nm (y-axis) for the ATCC strain and six clinically isolated strains (PA1–PA6) at concentrations of Ag NPs from 0.22 to 7.5 µg/ml (x-axis). ****< 0.0001, ***0.0001, **< 0.001, *< 0.01.

The microtiter plate assays showed that all strains were strong biofilm producers (see Supplementary Table 3 for more details). Ag NPs at concentrations ranging from 0.225 to 7.5 µg/ml were used to assess the antibacterial activity of Ag NPs against the PA strains (reference strain and six clinical strains) with resistance to various antibiotics. Despite the variation between different strains, incubating PA strains with Ag NPs in doses ranging from 0.22 to 7.5 g/ml had an adverse effect on growth (Fig. 3). At concentrations of 0.9–7.5 µg/ml, Ag NPs were found to generate a significant decrease in growth (P < 0.05) in four of the PA strains (ATCC, PA1, PA2, PA6). Strains PA3, PA4 and PA5 were significantly impacted at concentrations above 1.8 µg/ml (F (3.013, 11.08) = 61.93, P < 0.0001). Concentrations below this negatively affected growth in all strains, although this was not statistically significant. However, at 0.225 µg/ml, there was an increase in the growth of certain strains of PA (ATCC, PA1, PA5 and PA6).

**Figure 4:**
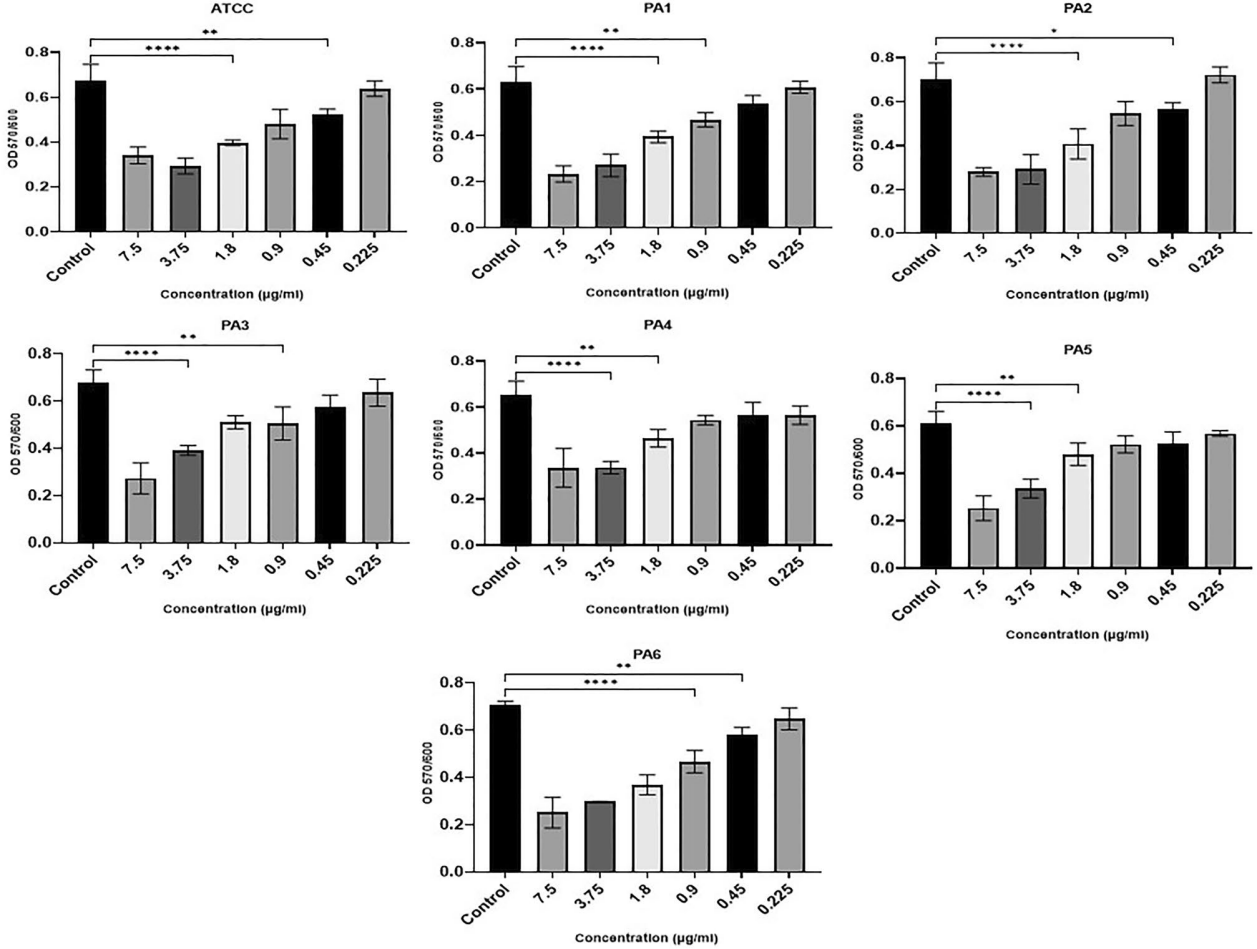
The impact of Ag NPs on biofilm development for the ATCC strain and six clinically isolated strains (PA1-PA6) after incubation for 24 h at Ag NP concentrations from 0.22 to 7.5 µg/ml (x-axis). ****< 0.0001, ***0.0001, **< 0.001, *< 0.01.

The biofilm development of three strains (ATCC, PA2, PA6) was significantly impacted by Ag NPs at concentrations at or above 0.45 µg/ml (F (1.931, 11.39) = 17.12, P = 0.0009). Only concentrations of 0.9 µg/ml and above substantially impacted PA1 and PA3. Concentrations of 1.8 µg/ml or over significantly reduced the biofilm formation of the more resistant strains of PA4 and PA5 (Fig. 4).

**Figure 5:**
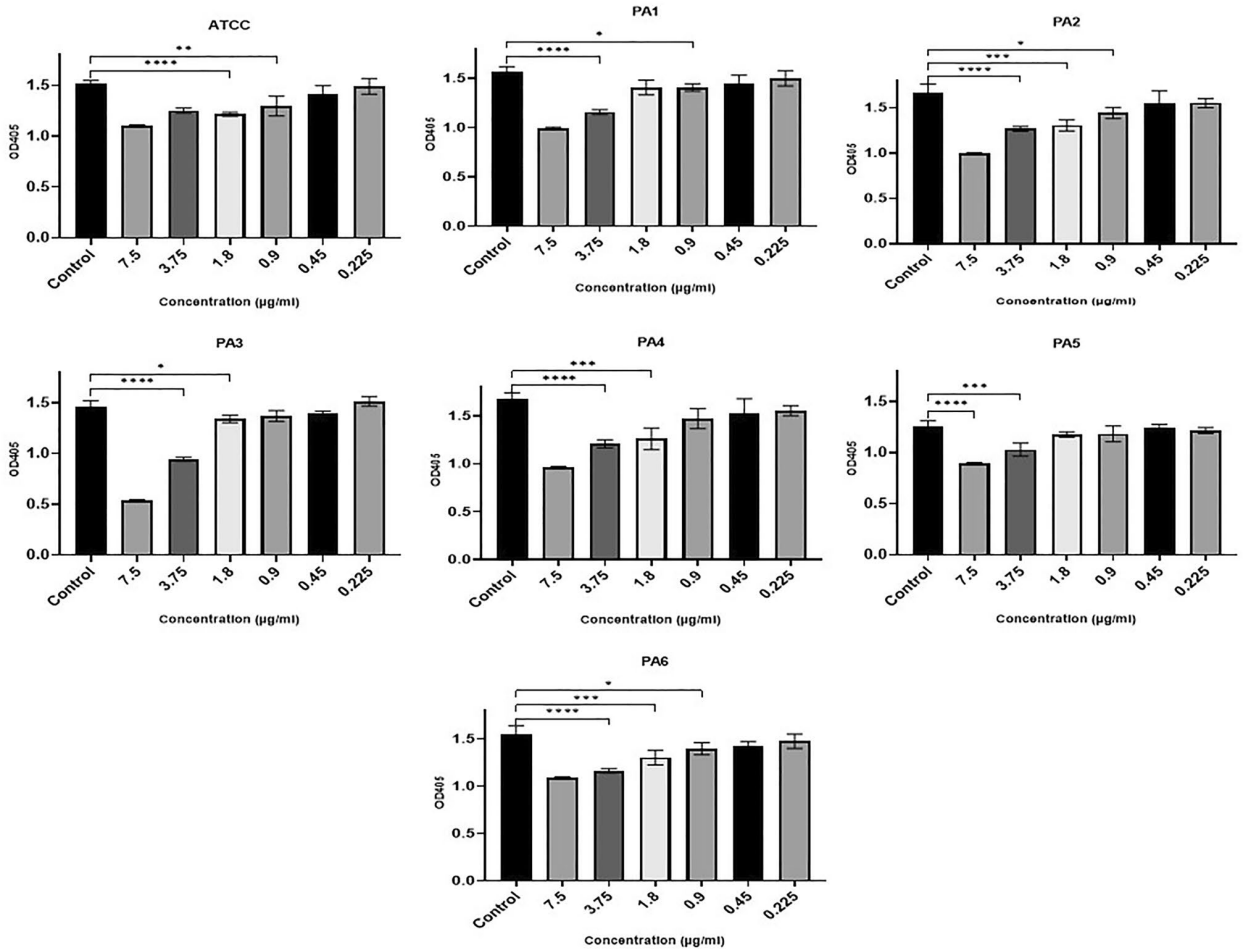
Ag NPs’ impact on the metabolic activity of PA planktonic cells, indicated by absorbance at 600 nm (y-axis) 4 h for the ATCC strain and six clinically isolated strains (PA1-PA6) at concentrations of Ag NPs from 0.22 to 7.5 µg/ml (x-axis). ****0.0001, ***0.0001, **< 0.001, *< 0.01.

Analysis of PA metabolism in biofilms revealed a statistically significant decline in PA levels in comparison to the control (F (2.757, 16.94) = 43.30, P < 0.0001) (Fig. 5). The biofilm cell metabolism of TCC, ATCC, PA1, PA2, and PA6, were significantly inhibited by 0.9 µg/ml Ag NPs. Significant effects on PA3 and PA4 were observed at concentrations of 1.8 µg/ml and higher. The PA5 strain was only found to be significantly impacted at concentrations above 3.75 µg/ml. Nonetheless, PA5’s biofilm cell metabolic activity was significantly impacted at all Ag NP concentrations.

In comparison to the hospital strains, the reference strain demonstrated greater sensitivity to all the Ag NP concentrations examined. The lower concentrations of Ag NPs needed to significantly impact the ATCC strain supports the findings of the antibiotic assays, where it was found to be sensitive to all the tested antibiotics.

### Effect of Ag NPs on EPS production

**Figure 6:**
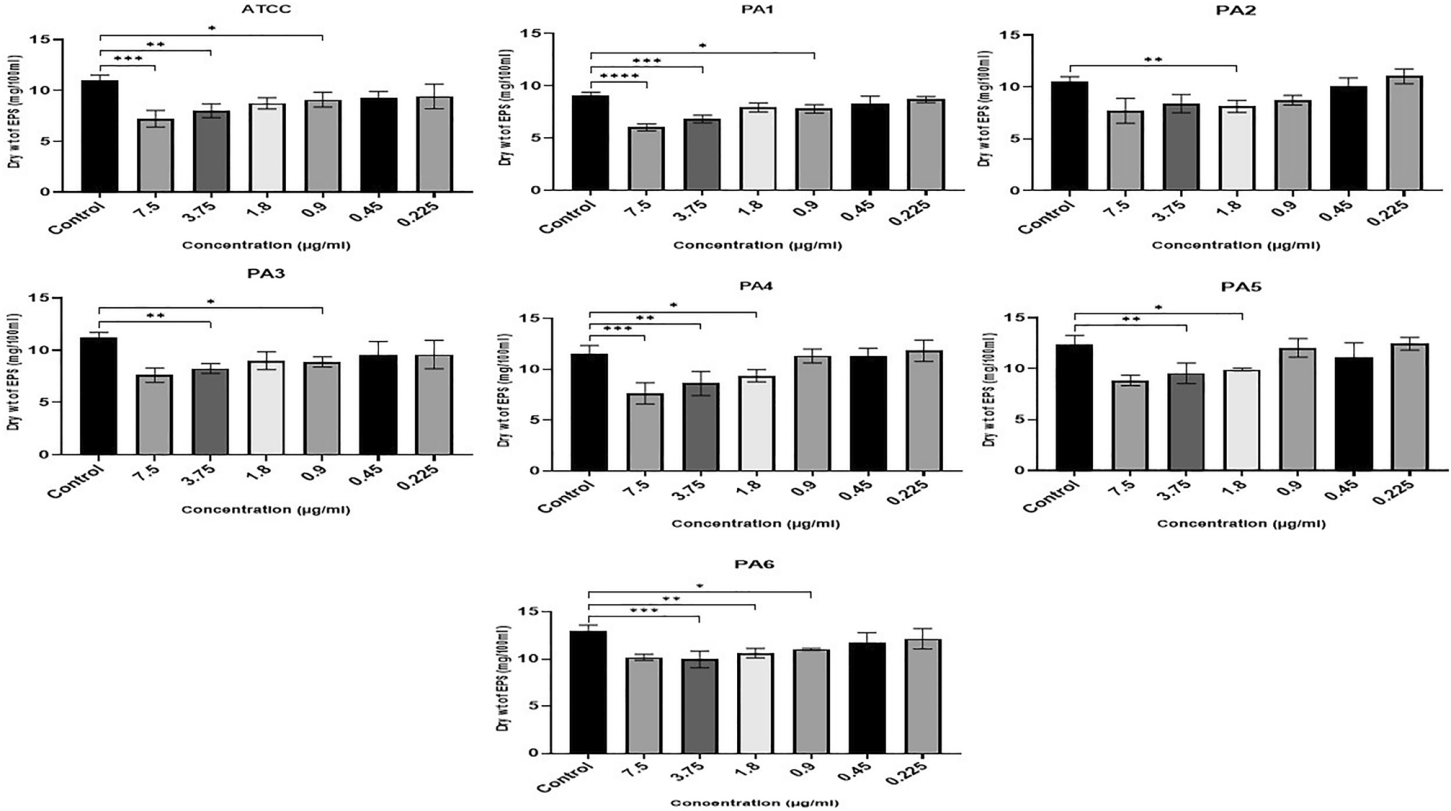
Ag NPs’ impact on biofilm EPS production, indicated by dry weight of EPS (mg/100 ml) for the ATCC strain and the six clinical isolates (PA1-PA6) at Ag NP concentrations from 0.22 to 7.5 µg/ml (x-axis). ****0.0001, ***0.0001, **< 0.001, *< 0.01.

The effect of Ag NPs on EPS production was examined by culturing the PA strains with no Ag NPs or with subinhibitory concentrations of Ag NPs. The dry weight of the EPS extracted after culture was significantly lower in the Ag NP-treated cells than the control cells (F (6, 42) = 8.38, P ≤ 0.0001). Figure 6 shows that the weight of EPS extracted from PA ATCC, PA1, PA3, and PA6 was significantly lower than that of the control when they were grown in the presence of Ag NP concentrations at or above 0.9 µg/ml. For PA2, PA4 and PA5 the reductions in EPS production were statistically significant at Ag NP concentrations at or above 1.8 µg/ml.

### Inhibitory effect of Ag NPs on established PA biofilm

**Figure 7:**
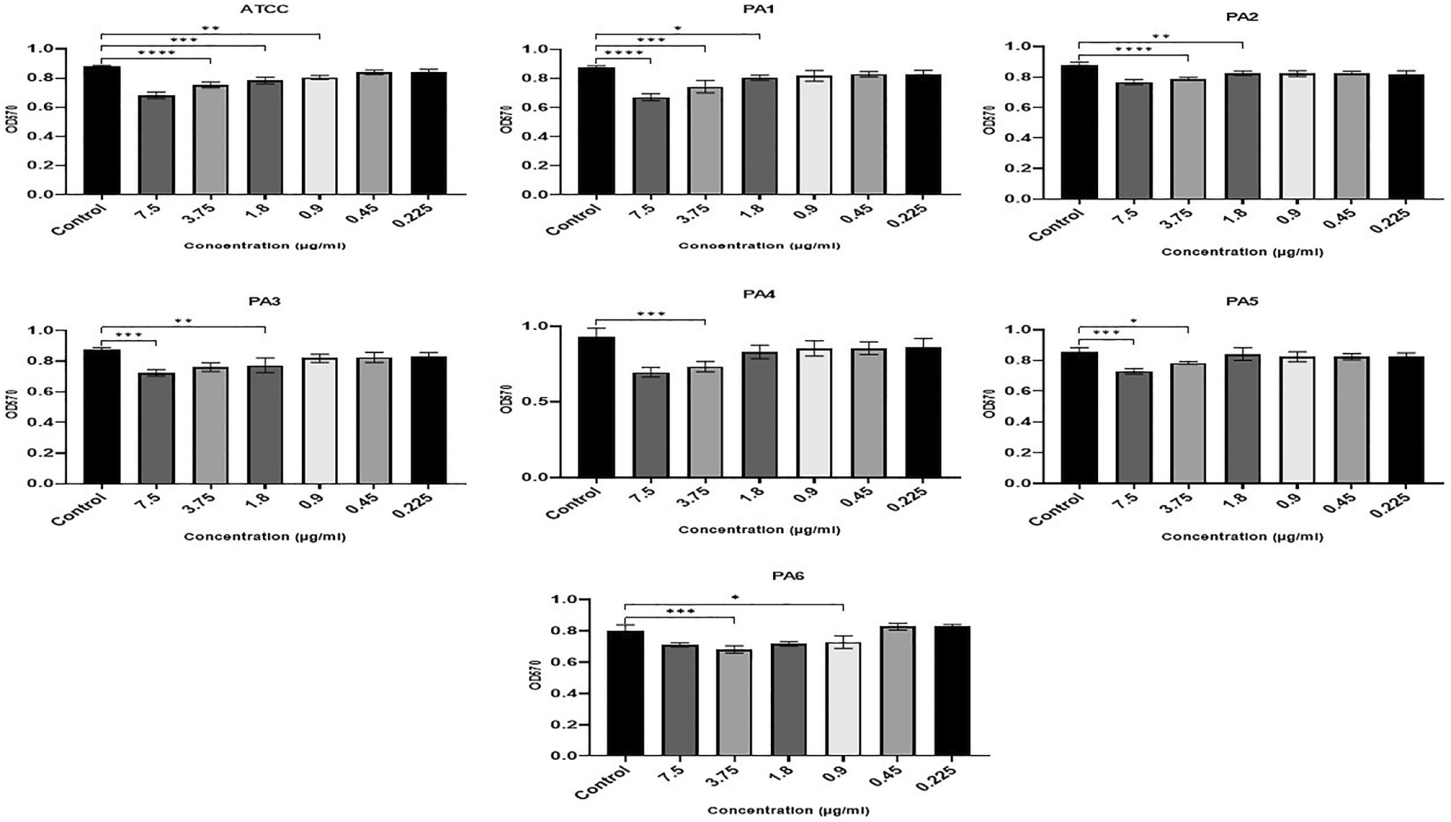
Ag NPs’ impact on the mature biofilm eradication following 24-h incubation at concentrations from 0.22 to 7.5 µg/ml (x-axis) denoted by OD at 570 nm (y-axis). (****0.0001, ***0.0001, **< 0.001, *< 0.01).

The results indicate that Ag NPs also significantly reduced CV-stained PA biomass in the eradication assays that evaluated the treatment of 1-day old pre-formed biofilms (F (20, 66) = 9.148, P = 0.0001). At the same time, Ag NPs inhibited the metabolic activity of mature biofilms. Figure 7 shows that two of the strains (ATCC, PA6) showed statistically significant decreases in CV staining biomass at concentrations above 0.9 µg/ml. Three strains (PA1, PA2, PA3) were also significantly inhibited by an Ag NP concentration of 1.8 µg/ml. The substantial CV staining of PA4 and PA5 was significantly reduced by concentrations above 3.75 µg/ml.

**Figure 8:**
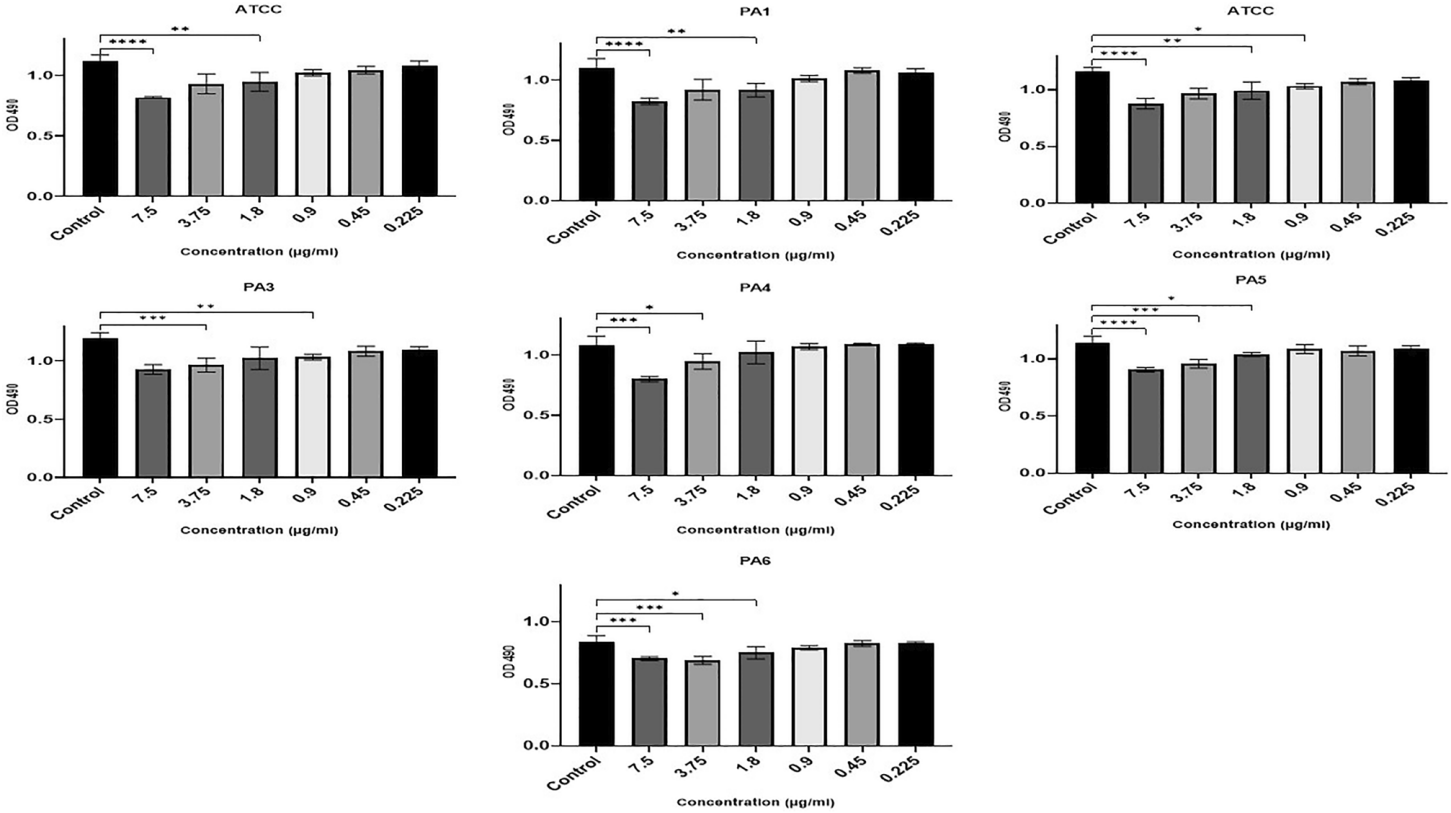
Ag NPs’ impact on the metabolic activity of mature biofilm cells using TCC staining following 24-h incubation with Ag NPs concentrations from 0.22 to 7.5 µg/ml (x-axis). This is denoted by OD at 570 nm (y-axis) (< 0.0001, ***0.0001, **< 0.001, *< 0.01).

The metabolism of the preformed biofilm community of PA strains incubated with Ag NPs were significantly inhibited (F (6, 7) = 15.32, P = 0.001). In strains PA2 and PA3, Ag NP concentrations of 0.9 µg/ml significantly inhibited metabolic activity, but concentrations of 1.8 µg/ml or above were needed to significantly inhibit the metabolic activity of ATCC, PA1, PA5, and PA6. Only concentrations of 3.75 µg/ml and higher significantly impacted PA4 metabolic activity (Fig. 8).

### The impact of Ag NPs on the expression of QS-regulated genes

**Figure 9:**
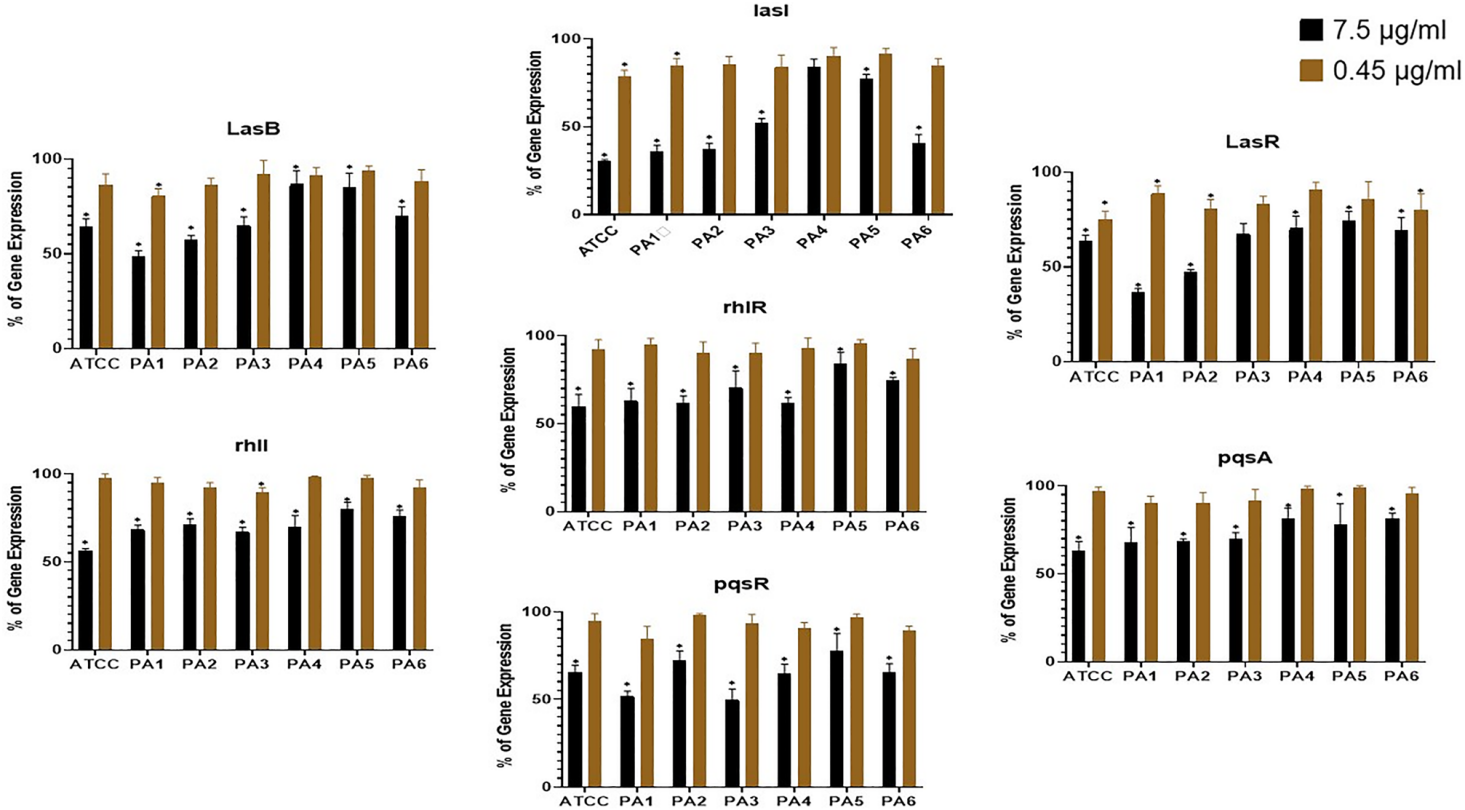
The effect of Ag NPs on the expression of QS-regulated genes in PA strains exposed to biosynthesized Ag NPs relative to the gene expression of controls not exposed to Ag NPs. Ag NP concentrations of 7.5 µg/ml and 0.45 µg/ml. *< 0.01.

Calculated Ct values were used to determine the corresponding expressions of the QS-regulated genes *lasI, lasR, rhlI, rhlR, pqsR, and pqsA*. The gene expressions were first standardized relative to the average of the *RopD* reference gene. Figure 9 compares the relative expression of QS-regulated genes in the PA strain exposed to biosynthesized Ag NPs (at concentrations of 7.5 µg/ml and 0.45 µg/ml) to the control samples cultured without Ag NPs. As Fig. 7 shows, Ag NPs reduce the expression of QS-regulatory genes. Compared to the control, where gene expression is assumed to be 100%, all but two PA strains cultured with 7.5 µg/ml Ag NPs for 24 h show significantly reduced QS-regulated gene expressions. The two exceptions (*LasR* in PA3 and *LasI* of PA4) showed reductions in expression but this was statistically insignificant.

The percentage of inhibition varied between strains and between different genes (Table 1). In comparison to the control sample, at 7.5 µg/ml Ag NPs, the relative expression of the *Las* gene system was markedly reduced in all strains. The decrease in *LasR*, *LasB*, and *LasI* was between 25.7 and 63.3%, 13.2 and 51.4%, and 15.7 and 69.4%, respectively. At 7.5 µg/ml concentrations, both *rhIR* and *rhII*'s gene expression showed statistically significant decreases, ranging from 19.7 to 43.8% and 16–40.3%, respectively. Additionally, at the high concentration the expression of *PqsA* and *PqsR* were substantially reduced compared to the controls.

**Table 1:** Percentage of inhibition of the relative expression of QS- regulatory gene compared to control (100%).

	LasR		LasB		LasI		rhII		rhIR		pqsA		pqsR	
	7.5 µg/ml	0.45 µg/ml	7.5 µg/ml	0.45 µg/ml	7.5 µg/ml	0.45 µg/ml	7.5 µg/ml	0.45 µg/ml	7.5 µg/ml	0.45 µg/ml	7.5 µg/ml	0.45 µg/ml	7.5 µg/ml	0.45 µg/ml
ATCC	36.5%	25.1%	35.7%	13.6%	69.4%	21.6%	43.8%	2.4%	40.3%	7.6%	36.9%	2.9%	34.8%	5.0%
PA1	63.3%	11.2%	51.4%	19.2%	64.3%	15.3%	31.7%	5.1%	37.0%	4.8%	32.3%	9.6%	47.9%	15.3%
PA2	52.4%	19.0%	42.8%	13.9%	63.0%	14.7%	28.6%	8.0%	38.0%	10.0%	31.5%	9.6%	27.9%	1.9%
PA3	32.4%	16.5%	34.9%	8.0%	47.7%	16.1%	32.7%	10.4%	29.5%	10.0%	30.2%	8.4%	50.2%	6.8%
PA4	29.6%	12.4%	13.2%	8.6%	15.7%	10.1%	30.0%	1.7%	38.6%	7.4%	18.3%	1.3%	35.0%	9.2%
PA5	25.7%	14.5%	15.1%	16.2%	22.7%	8.7%	19.7%	2.4%	16.0%	4.2%	22.1%	1.1%	22.4%	3.5%
PA6	30.8%	20.2%	30.1%	11.4%	59.1%	15.1%	24.3%	7.8%	25.2%	13.2%	18.5%	4.1%	34.7%	10.6%

At the 0.45 µg/ml Ag NP concentration, there were also some statistically significant effects on the relative expression of certain genes. *Las* system genes were the most affected, with the relative expression of the *LasR* gene statistically reduced in 4 strains (ATCC, PA1, PA2 and PA 6). *LasI* gene expression was significantly reduced in ATCC and PA1, while *LasB* only showed a significant reduction in PA1. There was no statistically significant impact of the 0.45 µg/ml Ag NP concentration on the *RhI* and *pqs* system apart from on *rhII* gene expression in PA3.

### Cytotoxicity of AgNPs

**Figure 10:**
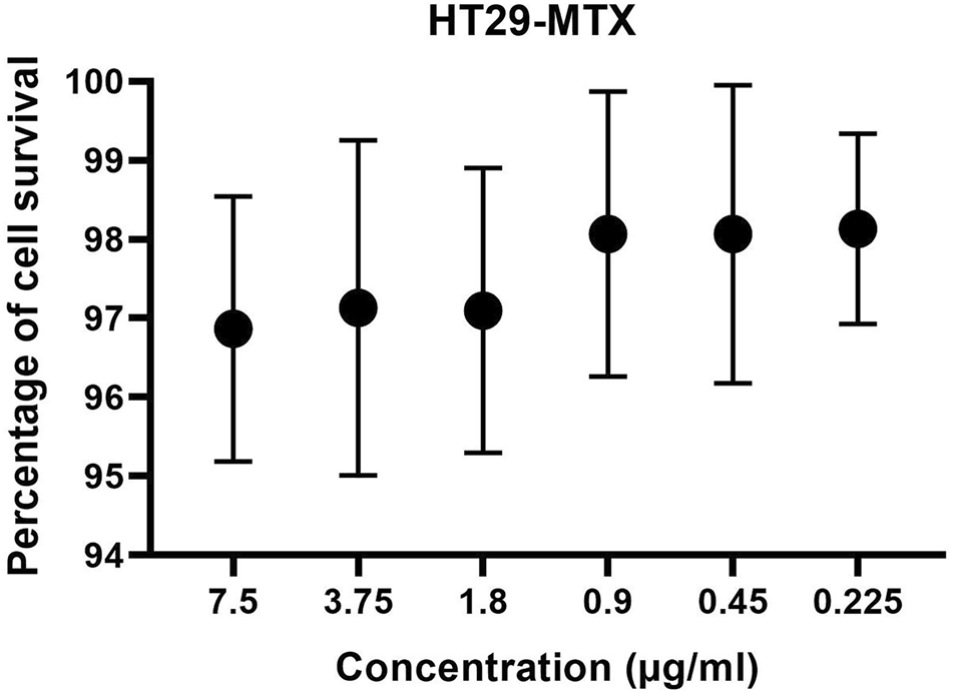
The influence of different concentration of Ag NPs on the viability of human HT29-MTX cell line after 24 h’ incubation. The relative cell viability was compared to the control (tested/control × 100). The experiment was performed 3 times in duplicates.

MTT assays were used for the initial screening of Ag NP cytotoxicity on the HT29-MTX cancer cell lines. HT29-MTX cell line showed a high resistance to Ag NPs at all concentrations used, with no statistically significant reductions in cell viability (F (6, 14) = 0.2871, P = 0.9334). Nonetheless, cell viability was reduced from 4.1% at an Ag NP concentration of 7.5 µg/ml to 0.2% at 0.225 µg/ml (Fig. 10).

## Discussion

Biofilm-forming microbes are responsible for many diseases. One study performed by the National Institutes of Health and Centre of Disease Control found that biofilm-forming microbes caused between 65 and 80% of infections^40^. Many studies have indicated that NPs serve as effective biofilm inhibitors against target bacteria^41,42^. Additionally, in previous work we found that ZnO nanoparticles played a critical role in counteracting the development of biofilm in biofilm-forming PA^43^. The current study focused on the use of *P. harmala* in Ag NP synthesis as this was thought to have both biological and economic benefits. The findings highlight the significant anti-QS, antibacterial, and antibiofilm properties of biosynthetic Ag NPs.

Chemical reduction is one of the processes utilized to create Ag NPs^44,45^. Reduction approaches are thought to be simpler and more affordable than other alternative methods^46^. Furthermore, it is strongly recommended that ecologically friendly techniques are used to synthesize Ag NPs (i.e., green synthesis approaches that involve both plants and microorganisms)^47^. In the current study, reducing agents from plant secondary metabolites were used to create the NPs^48^. Ag NPs have been biosynthesized using a variety of plants, and various biological processes have been assessed. In the present work, *P harmala* was used, a herb that has many phytochemical constituents that have been widely applied in traditional medicine^49^.

Our study revealed that during the biosynthesis, Ag NPs start to develop when silver nitrate is exposed to *P haramla* seed extract. This was confirmed through color changes and the use of UV–Vis spectroscopy. The biosynthesized Ag NPs showed a strong absorption band at 461 nm as a result of their SPR characteristics. The reduction and stability of silver nanoparticles may be caused by interactions between silver atoms and bioactive chemicals^50^, and these were investigated using FT-IR analysis. Similar peaks were evident in the FT-IR spectra of the *P. harmala* extract used to synthesize the Ag NPs, although there were also small shifts in both spectra. The spectra of the seed-extract-based Ag NPs revealed a range of absorption bands between 1642 and 3351 cm^−1^. This indicates that the biomolecule has the capacity to reduce and to stabilize silver ions (Ag^+^, Ag^0^) in Ag NPs when placed in aqueous seed extract.

Additional TEM detection revealed that these particles were roughly spherical in shape and were uniformly sized, with an average diameter of 11 nm. Ag NPs' antibacterial properties are negatively correlated with their particle size, being weaker the larger the diameter^51^. According to Choi et al.^52^, Ag NPs smaller than 15 nm can enter the bacterium and exert substantially more potent antibacterial effects than those sized 15–20 nm. Particles of 1–15 nm are able to adhere to the bacteria's surface.

We used our Ag NPs to test their bactericidal efficacy against PA. Our findings demonstrated that Ag NPs had a potent antibacterial effect on PA at low concentrations, with the MIC and MBC between 15.6 and 31.25 µg/ml, respectively. The Ag NPs’ MIC for PA has been reported to be from 0.59 to 50 µg/ml^53–60^. The wide range of MIC values found in different studies is because differences in the nanoparticle's physicochemical properties (i.e., shape, size and presence of surface moieties) have a substantial impact on their antibacterial activity^51^. Our Ag NPs’ MIC is consistent with the lower range of known MICs, and this is probably due to their relatively small particle size. We were therefore able to efficiently prevent the growth of PA using low concentration of Ag NPs. The inhibitory effect of Ag NPs on *Escherichia coli* (*E. coli*) and *Staphylococcus aureus* (*S. aureus*) were studied by Azizi et al.^60^, who also synthesized them using *P. harmala*. While the Ag NPs produced were larger (23 nm), they nonetheless showed a moderate inhibitory action—again demonstrating the effectiveness of Ag NPs against pathogenic bacteria. However, the Ag NP’s effects on PA was not investigated by these authors.

The microtiter plate test was also carried out in this study to examine the inhibition of biofilm formation in PA in the presence of different concentrations of Ag NPs. The findings showed that there was indeed a statistically significant inhibition of biofilm formation (Figs. 3, 4) as well as a reduction in EPS (Fig. 6). Our findings are in line with earlier studies carried out by Haidari et al.^61^, who investigated the impact of Ag NPs on the biofilm production in various bacteria and discovered that Ag NPs at a 45 µg/ml concentration can inhibit the biofilm formation of *S. aureus* by 89% and of *E. coli* by 75%.

The effectiveness of Ag NPs (sized 7–70 nm) against PA ATCC strain PAO1 was also demonstrated by Loo et al.^62^, who showed the breakdown of the EPS matrix resulted in a 95% reduction in the generation of biofilm by PA. Ag NPs can cause significant structural damage to bacterial biofilms. This can be seen in the changed morphology of the biofilms resulting from cell lysis^14^, cell wall damage, and the disruption of membrane corrugation and membrane polarization/permeability. Particular strains of bacteria also form specific EPS matrices that enclose them^15^. The Ag NPs interact electrostatically with bacterial membranes and this can be strong enough to rupture the membranes and allow the NPs to penetrate the mature biofilm. A study carried out by Haney et al.^63^, however, was contrary to our findings and found that treatment of cells with superparamagnetic iron oxide nanoparticles at a dosage of 200 µg/ml increased the biomass of the PA biofilm. This effect was explained by the fact that cells may employ iron nanoparticles to provide elemental iron, leading to the observed increase in cell density and biofilm biomass development—a process not available in the case of Ag NPs.

In our study, Ag NPs were able to reduce the biomass of established biofilms. However, higher concentrations are required to achieve a statistically significant reduction than that needed to significantly impact early biofilm formation—reinforcing the view that biofilms’ primary function is protective^64,65^. According to Said et al.^65^, when the biofilm matrix is broken up by ethylenediaminetetraacetic acid (EDTA) and benzethonium chloride (BC), the biofilm cells are much more susceptible to Ag NPs (as measured through microcalorimetry).

Our findings showed that Ag NPs reduced the metabolic activity of PA in mature biofilm, which is in line with the mechanisms of action put forward by Li et al.^66^ and Kim et al.^67^. Ag NPs have the capacity to enter cells and pass through their peptidoglycan, periplasm, and outer membranes. This is where they destroy respiratory chain dehydrogenases and change the dissolved oxygen level^66,67^. Additionally, interactions could take place between Ag^+^ and the thiol (–SH) group of cysteines to generate –S–Ag. In turn, this would inhibit the development and suppress the enzymatic activity of the bacterial cells^66,68^.

Bacterial pathogenicity and biofilm-forming capacity are controlled by the QS network^69^, which contains transcriptional regulators activated by their natural autoinducers (i.e., *las* and *rhl*^22^). In addition to regulating virulence traits, QS also manages PA biofilm-forming capacity. Studies by Solano et al.^70^ and Brindhadevi et al.^69^ showed that PA with mutated *lasR* and *lasI* create biofilms that are less effective and can be easily removed by antimicrobials. Exopolysaccharides and other substances that regulate the shape of growing communities are encoded by genes that QS activates^40^.

A number of systems play a role in co-regulating biofilm formation in the overall PA system, including the *las*, *rhl*, and *pqs* systems. While *LasI* and *RhlI* regulate autoinducer synthesis, *lasR* and *rhlR* are primarily involved in coding transcriptional activators^71^. Ag NPs may interfere with the *las* and *rhl* pathways and prevent the production of signaling molecules, preventing the formation of biofilms in PA due to the anti-QS impact^64,72^. In the present work, when bacterial cells were treated with the Ag NPs, the expression of many important genes of the *las*, *rhl*, and *pqs* QS systems (*lasR*, *lasI*, *lasB*, *rhlI*, *rhlA*, *pqsR*, and *pqsA*) was significantly decreased compared to the control (Fig. 9 and Table 1).

It was suggested that Ag NPs may disrupt the primary QS system of PA. Nonetheless, it is important to note that the development of biofilms is a dynamic process, and QS production follows irreversible bacterial attachment as well as post-inoculation cell multiplication and expansion. As a result, during the first stages of biofilm development, Ag NPs’ inhibitory impact on biofilm biomasses through interference with the QS system was distinct at early biofilm formation at lower Ag NP concentrations than that needed to effect the established biofilm.

Currently, it is believed that Ag NPs work by disrupting the bacterial cell walls’ integrity and membrane, increasing the permeability of the membrane and promoting cell death^73^. Moreover, the Ag NPs disrupt the respiratory chain reaction by combining with sulfhydryl groups, which causes lipid peroxidation and facilitates oxidative damage to DNA and relevant proteins, after which cell death occurs^74,75^. Ag NPs also adhere to sulfurous and phosphorous DNA groups, which can ultimately damage the DNA and interfere with its transcription and translation^76^. Additionally, Ag NPs interrupt cell signal transduction and initiate cell death through the dephosphorylation of phosphotyrosines^77^. When Ag NPs are exposed to aerobic conditions, they may release Ag^+^ from the particles’ surface. The released Ag^+^ has a significant antimicrobial effect as it interacts with the cell walls and membranes of the bacteria. This is one of the key reasons for Ag NPs’ toxicity^78^.

Even though Ag NPs have great potential to serve as antimicrobial agents, there are concerns regarding their safety in humans, and the development of silver resistance in bacteria, including *Pseudomonas*. The cytotoxic potential of Ag NPs has hindered their establishment as promising chemotherapeutic agents. Siddique et al.^79^ examined the toxic potential of Ag NPs against HeLa cell lines at various concentrations using the neutral red uptake test. Their results found that these NPs were nontoxic up to 120 μg/ml concentrations, and a statistically significant cytotoxic effects occur at a concentration of 240 μg/ml—a higher concentration than used in our present work. Additionally, the toxic concentration of Ag NPs range from 10 to 100 µg/ml in studies that have assessed the impact of Ag NPs on human cell culture in vitro^80–82^. In our work, statistically significant inhibition was not found when we challenged HT29-MTX cell line with the biosynthesized Ag NPs at similar concentrations, suggesting that the Ag NPs could be safe if used in low amounts.

The emergence of bacterial resistance to silver through bacteria’s horizontal gene transfer^83^ is a serious concern that has arisen from the increasing use of silver in medicine and industry, as well as in domestic antimicrobials. Some studies have shown PA^84^ and other species of bacteria^85,86^ to be able to reduce soluble Ag^+^ to colloidal Ag or NP Ag^0^, through an as yet unknown reduction process^12^. Muller and Merrett^86^ have proposed that Ag^+^ is removed by bacteria from the surrounding environment before it can penetrate the cell, thereby lowering the need for protein-silver binding and cell efflux processes. The destructive and inhibitory effect of silver on biofilms does not fully prevent the biofilms adapting to silver in both NAg and ionic Ag^+^ forms. Nonetheless, compared to commonly used antibiotics, silver remains an effective antibiofilm agent^87,88^.

When examining the bactericidal impact of Ag NPs, certain PA strains showed an unexpected increase in growth at the lower concentrations (Fig. 3). In earlier work, AgNO3 concentrations below bactericidal levels (3–8 µg/l) were found to stimulate, rather than inhibit, the growth of *E. coli*^89^. Similar findings were obtained by Schacht et al.^90^, where Ag NPs less than 15 nm and at concentrations between 20 and 60 µg/ml were found to stimulate the growth of some bacteria. Ag NPs sized 2.8–10.5 nm and coated with polyethylene glycol were shown by Xiu et al. ^89^ to increase *E. coli* K12 growth from 6 to 13% at concentrations of 1.8–2.2 µg/ml. The authors also found Ag NPs sized 20–80 nm and coated with polyvinyl pyrrolidone to increase the growth of *E. coli* from 11 to 21% at concentrations of 5.7–16.4 µg/ml.

Increased or stimulated growth of organisms under sub-lethal exposure to inhibitory agents is a biphasic dose response known as the hormetic response. The characteristic response pattern of microbes is of stimulated growth at low concentrations of the inhibitor and inhibited growth at high concentrations ^91^. Studies of antibiotic effects on bacteria that have seen hormetic responses have concluded that they represent a novel survival mechanism that can lead to exponential growth in the presence of low antibiotic concentrations. Hormetic responses have also been studied in relation to NPs^87,92^, but currently how they are achieved in bacterial cells is poorly understood. The current authors have attempted to explain these unexpected enhancements of growth by reference to a non-genetic process known as ‘persistence’.

This mechanism involves subpopulations of cells (persister cells) that are less affected by antibacterial agents and therefore survive longer than other more susceptible subpopulations ^93^. In environments with low concentrations of Ag NPs (Ag^+^), the defence mechanism of the bacterial cells has sufficient time to adjust to the toxic effects of Ag NPs. The DNA-repair regulatory network's ‘SOS’ response ^94^ is then triggered by the buildup of aberrant single-stranded DNA inside the cell caused by the geno-toxic effects of Ag NP.

Our study revealed that Ag NPs can reduce growth, prevent biofilm formation, and eliminate established PA biofilms. These findings highlight the promising future of Ag NPs to be used as alternative antimicrobial agents or in conjunction with antibiotics. The development of silver resistance, however, remains a concern. Therefore, it is recommended that research focuses on examining the combined action of Ag NPs and antibiotics to understand how they work against different microorganisms, especially resistant hospital strains. In time, Ag NPs may become an effective alternative therapy for combatting infections. Strategic control and treatment of biofilm infections will be made easier as our understanding of the mechanism for biofilm formation increases. The QS signaling cascade affects a variety of bacterial activities, including pathogenicity and biofilm formation^95^. By blocking the QS cascades, bacterial pathogenicity and virulence can be decreased. Further research is required to confirm the molecular mechanism of Ag NPs and QSI in preventing PA biofilm development.

## Supplementary Information

Supplementary Information.

## Data Availability

All data generated or analysed during this study are included in this published article and its supplementary information files.
