# Supplementary material for: The efficacy of biosynthesized silver nanoparticles against Pseudomonas aeruginosa isolates from cystic fibrosis patients
Source: Sci Rep. 2023 Jun 1;13:8876. doi: 10.1038/s41598-023-35919-6 (PMC10235065; doi:10.1038/s41598-023-35919-6)
Supplement: Supplementary file 1 — Supplementary Information. [file 41598_2023_35919_MOESM1_ESM.pdf]

The Efficacy of Biosynthesized Silver Nanoparticles Against Pseudomonas aeruginosa Isolates from Cystic Fibrosis Patients

Hafez Al-Momani[a], Muna Almasri[b] , Dua'A Al Balawi[b], Saja Hamed[c], Borhan Aldeen Albiss [d],Nour Aldabaibeh[e] Lugain Ibrahim[b], Hadeel Al Balawi[b], Sameer Al Haj Mahmoud[f], Ashraf I. Khasawneh[a], Muna Kilani[g] ,Muneef alduferi[h], Muayyad Bani-Hani[i], Matthew Wilcox[h], Jeffrey Pearson[h], Christopher Ward[h].

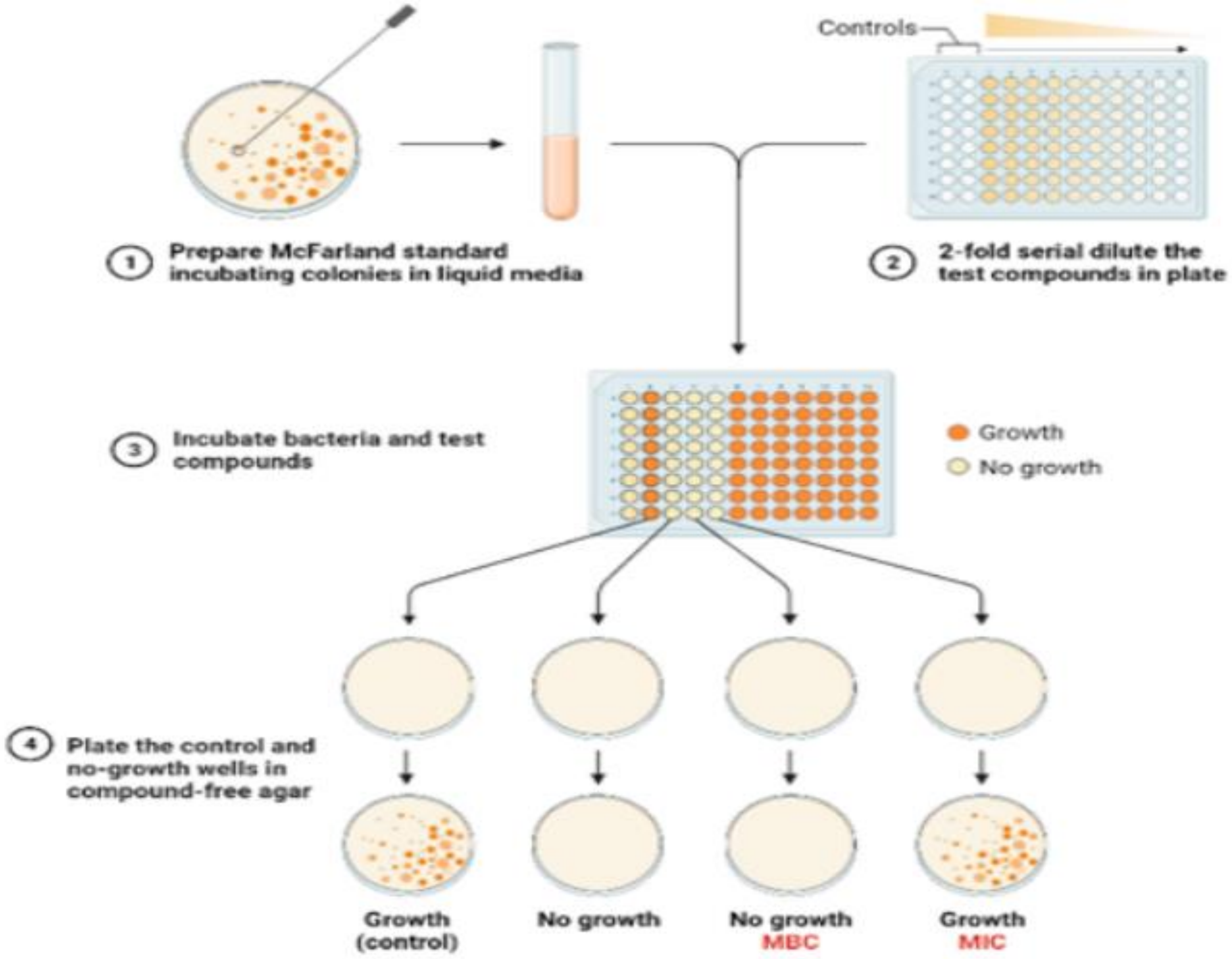

Supplementary figure 1: Broth Micro-dilution method for MIC and MBC determination

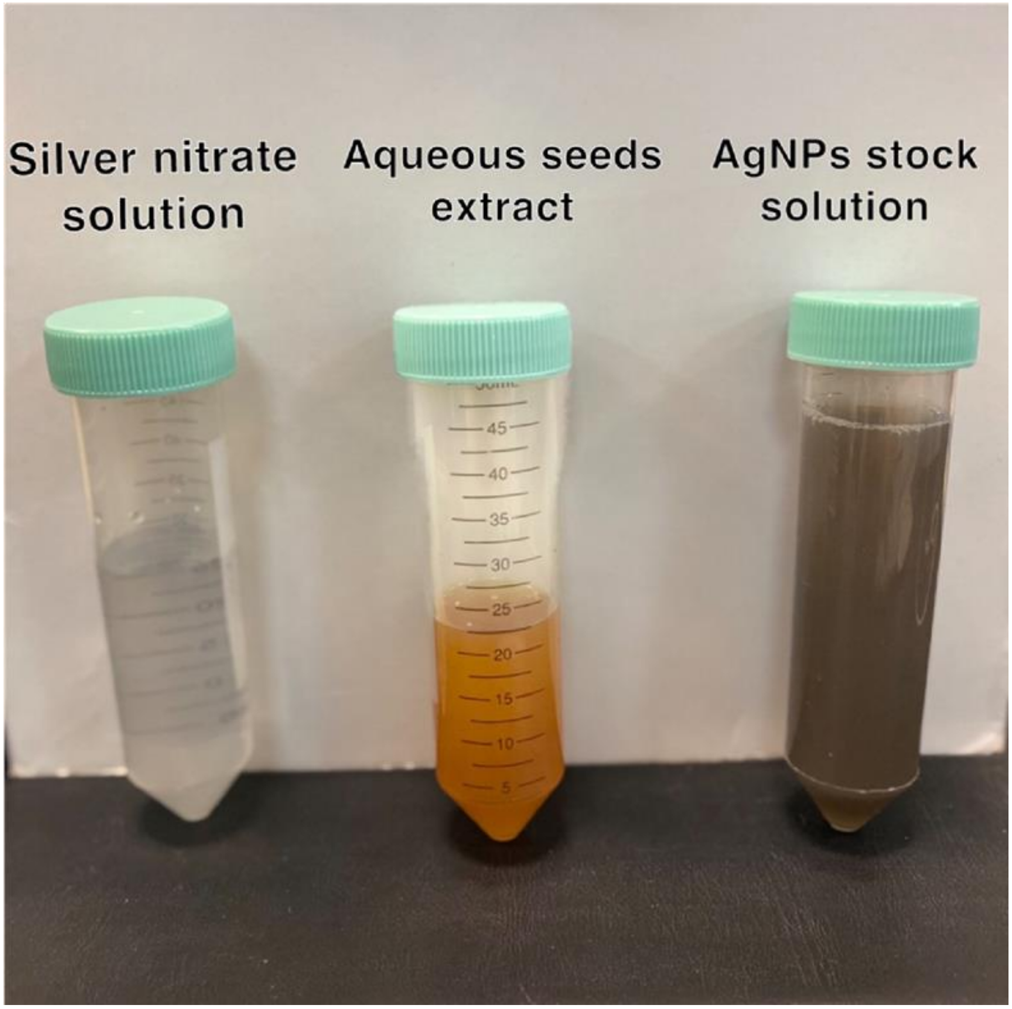

Supplementary Figure 2: The color-changing indicators that appear during Ag NPs synthesis.

Gene		Primer sequence
ropD	F	5′-CGAACTGCTTGCCGACTT-3′
	R	5′-GCGAGAGCCTCAAGGATAC-3
lasI	F	5′-CGCACATCTGGGAACTCA-3′
	R	5′-CGGCACGGATCATCATCT-3′
lasR	F	5′-CTGTGGATGCTCAAGGACTAC-3′
	R	5′-AACTGGTCTTGCCGATGG-3′
rhlI	F	5′-GTAGCGGGTTTGCGGATG-3′
	R	5′-CGGCATCAGGTCTTCATCG-3′
rhlR	F	5′-GCCAGCGTCTTGTTCGG-3′
	R	5′-CGGTCTGCCTGAGCCATC-3′
pqsA	F	5′-GACCGGCTGTATTCGATTC-3′
	R	5′-GCTGAACCAGGGAAAGAAC-3′
pqsR	F	5′-CTGATCTGCCGGTAATTGG-3′
	R	5′-ATCGACGAGGAACTGAAGA-3′
lasB	F	5′-GGAATGAACGAAGCGTTCTCCGAC-3′
	R	5′-TGGCGTCGACGAACACCTCG-3′

Supplementary table 1: Primers sequence used in this reaserch[1-3]

	Piperacillin (PRL)	Cefepime (FEP)	Ceftazidime (CAZ)	Aztreonam (ATM)	Imipenem (IPM)	Meropenem (MEM)	Gentamicin (CN)	Amikacin (AK)	Levofloxacin (LEV)	Ciprofloxacin (CIP)
ATCC	29 (S)	29(S)	29(S)	30(S)	21(S)	29(S)	20(S)	24(S)	26(S)	34(S)
PA1	42(S)	36(S)	34(S)	40(S)	35(S)	44(S)	20(S)	19(S)	44(S)	48(S)
PA2	32(S)	28(S)	29(S)	30(S)	25(S)	36(S)	14(I)	19(S)	32(S)	36(S)
PA3	33(S)	14(R)	28(S)	34(S)	36(S)	34(S)	17(S)	19(S)	23(S)	28(S)
PA4	25(S)	25(S)	25(S)	24(S)	18(I)	35(S)	29(S)	29(S)	21(I)	40(S)
PA5	29(S)	14(R)	26(S)	25(S)	32(S)	18(I)	14(I)	16(I)	20(I)	24(I)
PA6	28(S)	16R	24(S)	23(S)	17(I)	19(S)	13(I)	18(S)	16(I)	22(I)

Supplementary table 2: Interpretavie categories and zone Diameter breakpoint, nearest whole mm for PA ATCC 27853 and the six clinical strains, Abbreviations: ATCC, American type culture collection, I , intermediate, R resistance, S susceptible

	**Odc**	**OD of positive control**	**2*Odc**	**4*Odc**	
ATCC	0.09	1.01	0.18	0.36	strong-biofilm producer
PA1	0.06	0.475	0.12	0.24	strong-biofilm producer
PA2	0.06	0.764	0.12	0.24	strong-biofilm producer
PA3	0.07	0.627	0.14	0.28	strong-biofilm producer
PA4	0.08	0.676	0.16	0.32	strong-biofilm producer
PA5	0.07	0.642	0.14	0.28	strong-biofilm producer
PA6	0.06	0.712	0.12	0.24	strong-biofilm producer

Supplementary Table 3: Biofilm characteristics of the strain used in this study
